# Exploring reference ranges for thyroid-stimulating hormone in neonatal screening tests for preterm infants: a 5-year retrospective study

**DOI:** 10.3389/fped.2026.1781279

**Published:** 2026-04-08

**Authors:** Xing Huang, Fei Xiong, Shulan Zhu, Fan Yang

**Affiliations:** 1 Department of Pediatrics, West China Second University Hospital, Sichuan University, Chengdu, China; 2 Key Laboratory of Birth Defects and Related Diseases of Women and Children (Sichuan University), Ministry of Education, Chengdu, Sichuan, China

**Keywords:** birth weight, gestational age, postnatal age, preterm infants, small for gestational age, thyroid function, thyroid-stimulating hormone

## Abstract

**Purpose:**

Currently, the same thyroid-stimulating hormone (TSH) cutoffs are used for term and preterm infants. Our objective was to determine TSH reference ranges and cut-off values for preterm infants born at different gestational ages (GA), birth weight (BW), and postnatal age (PNA).

**Methods:**

A retrospective analysis was performed on preterm infants who received neonatal blood screening tests (NBST) between October 2017 and December 2022. Newborns were classified based on GA, BW, and small-for-gestational-age (SGA) status. For each category, the 2.5th, 5th, 50th, 95th, and 97.5th percentiles of TSH levels were determined. The 97.5th percentile was established as the cutoff value for TSH screening. TSH reference thresholds were then established for different PNA periods.

**Results:**

Infants were categorized based on GA, BW, and SGA status. Through this categorization, it was discovered that there were disparities in the cut-off values of TSH among different groups. In particular, during the first four weeks after birth in preterm infants, the 97.5th percentile TSH level (screening cutoff), one of the key indicators in NBST, shows a sequential trend of initial decline, subsequent rise, and final decrease, which peaked at 7.38 μIU/mL in the third postnatal week.

**Conclusion:**

TSH levels in preterm infants are influenced by GA, BW, and PNA, with SGA infants showing significantly higher TSH levels (*P* < 0.05). Developing condition-specific TSH reference ranges is essential for improving congenital hypothyroidism (CH) diagnosis and management.

## Introduction

Due to the immaturity of the hypothalamus-pituitary-thyroid (HPT) axis and disease factors, preterm infants are more prone to thyroid dysfunction (1, 2). In recent years, congenital hypothyroidism (CH) has been diagnosed with increased frequency in infants born preterm (3). The neonatal blood screening tests (NBST) program uses thyroid-stimulating hormone (TSH) levels to detect CH in preterm infants. Differences in thyroid axis physiology exist between term and preterm-born infants during the first month of life (4). TSH levels in preterm infants are influenced by a variety of factors, including gestational age(GA), Birth weight (BW), sex, postnatal age (PNA), and whether they were SGA (2, 5–10).

However, despite differences in GA, BW, and PNA-dependent thyroid hormone physiology (8, 11–16), the same TSH cutoffs are used for both preterm and term infants. Using TSH cutoffs derived from term infant data could lead to overdiagnosis or underdiagnosis of CH among preterm infants. Data on TSH reference ranges in preterm infants at different GA, BW, and PNA in China are limited.

Our objective for the current study was to determine TSH reference ranges for preterm infants born in different GA, different BW, and different PNAs, by analyzing a 5-year large birth cohort of more than 10,000 preterm infants in the western region of China.

## Subjects and methods

### Study population

**Figure 1:**
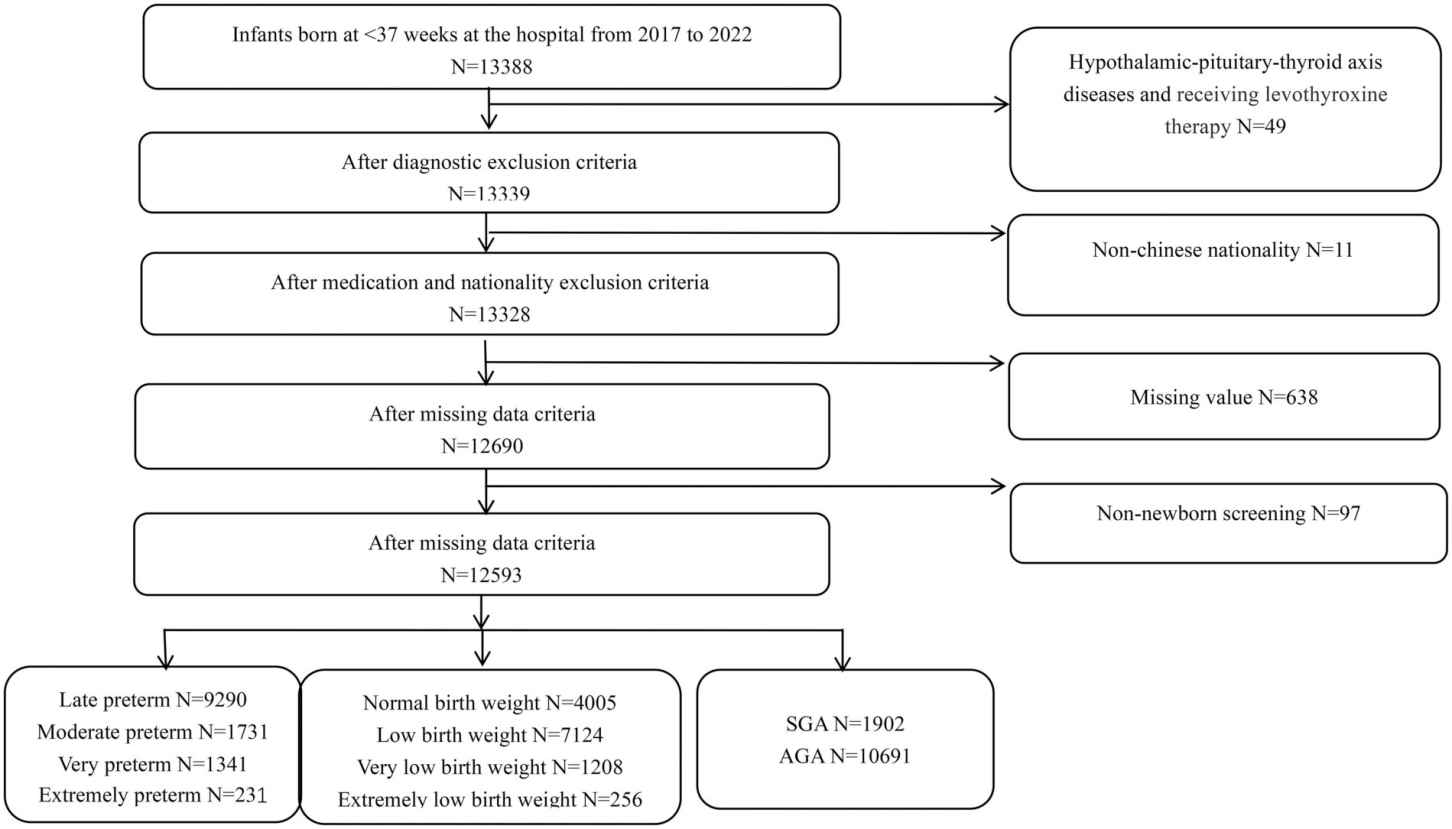
Flowchart of study participants.

This retrospective study collected data from the Children's Health Department of West China Second University Hospital, Sichuan University. Inclusion criteria: (1) Infants born at <37 weeks GA from October 2017 to December 2022; (2) Infants who underwent heel-stick NBST for TSH levels during this period. Exclusion criteria: (1) Infants diagnosed (based on ICD-10 codes and textual descriptions in discharge summaries) with HPT axis disorders and receiving levothyroxine therapy, including CH, transient hypothyroxinemia of prematurity (THOP), and hyperthyrotropinemia. (2) Infants whose mothers were non-Chinese nationals; (3) Infants lacking core data such as TSH values; (4) Infants not screened for TSH during the neonatal period (Figure 1).

The Ethics Committee of West China Second University Hospital, Sichuan University, approved the project as having no more than minimal risk and agreed to waive informed consent. The detailed population inclusion process is shown in Figure 1.

### TSH measurements

This study employed time-resolved fluorescence immunoassay (TRFIA) to measure TSH levels in neonatal heel-prick dried blood spot specimens. This method utilizes whole blood samples from peripheral blood; this non-radioactive, micro-immunoassay method offers high sensitivity and specificity, making it suitable for large-scale neonatal screening. Newborns with abnormal screening results require periodic thyroid function testing using chemiluminescent immunoassay (CLIA). This method utilizes venous serum; it offers higher sensitivity and superior precision in the low-concentration range. It is the preferred method for clinical confirmation of thyroid function, though it entails relatively higher testing costs and stricter instrument requirements, which makes it more suitable for precision testing in small samples.

### Study design

TSH screening is a routine part of the NBST program. The initial rapid screening using TRFIA was typically performed via heel-stick blood collection within 72 h to 7 days after birth. The TSH cutoff value used for NBST in our institution for both preterm and full-term infants is 9 μIU/mL. Infants with a GA of less than 32 weeks, a BW below 1,500 g, or high-risk factors for thyroid dysfunction required a second screening test (CLIA) using venous blood within 2 weeks after birth or 2 weeks after the initial screening test. Thyroid screening is regularly conducted in newborns with abnormal TSH test results. The TSH laboratory results employed in this study were obtained from routine clinical care. The NBS technique allows for blood collection after 72 h postpartum, following adequate breastfeeding. In our research, most infants had blood drawn between 72 h and 7 days after birth. A minority had blood drawn earlier or later due to complications, early discharge, parental request, or clinical indications. Samples collected at 2–4 weeks postpartum were not from follow-up or retesting of TSH-positive infants.

The central 95% (2.5th-97.5th percentile) interval, including the median, and the percentiles of 2.5th, 10th, 25th, 75th, and 97.5th, were determined for TSH, with the 97.5th percentile serving as the cutoff value. Infant demographics encompassed sex, maternal age, ethnicity, and the occurrence of multiple births and spontaneous pregnancy. In our study, preterm infants were categorized according to GA into overall preterm (less than 37 weeks), late preterm (34 to less than 37 weeks), moderate preterm (32 to less than 34 weeks), very preterm (28 to less than 32 weeks), and extremely preterm (less than 28 weeks). According to BW, they were further divided into four categories: normal birth weight (NBW, ≥2,500 g), low birth weight (LBW, 1,500 to less than 2,500 g), very low birth weight (VLBW, 1,000 to less than 1,500 g), and extremely low birth weight (ELBW, <1,000 g). All preterm infants were subsequently categorized into two groups: small for gestational age (SGA) and appropriate for gestational age (AGA). AGA was defined as newborns whose birth weight or length fell within the 10th to 90th percentile range corresponding to their gestational age and sex. SGA was defined as newborns whose birth weight or length was below the 10th percentile value corresponding to their gestational age and sex. The birth weight and length percentiles used in this study were based on the national growth reference standards for Chinese newborns by gestational age, as reported by Li and colleagues in the Chinese Journal of Pediatrics (2020) (17). These standards were developed using a large national sample and have been officially issued as the health industry standard of China (WS/T 800–2022). TSH reference ranges were calculated independently for the 95% central interval and for different postnatal blood collection time points.

### Statistical analysis

In this study, continuous variables were expressed as means and standard deviations (SD), or medians and interquartile ranges (IQR). Categorical variables were represented by frequencies and percentages. For continuous variables, the Kruskal–Wallis H test was used for one-way tests; for categorical data, the chi-square test was employed. We calculated crude and multivariable-adjusted relative risks (RR) and 95% confidence intervals (CI) using generalized linear model (GLM) with Poisson regression, by integrating univariate and multivariate analyses to assess the effects of GA, BW, and other factors on TSH levels. Statistical significance was assessed using a two-tailed test, with a significance level set at a *P* value of < 0.05. We analyzed the data using R version 4.0.2 (Comprehensive R Archive Network: http://cran.r-project.org).

## Results

### Demographic characteristics

From 2017 to 2022, our institution provided NBS to 13,388 preterm infants with a GA of less than 37 weeks. A total of 795 cases were excluded based on predefined exclusion criteria, including 49 infants with HPT axis disorders receiving levothyroxine therapy (37 with CH, 12 with hyperthyrotropinemia), 11 infants born to non-Chinese mothers (2 from South Korea, 3 from Australia, 1 from Pakistan, 1 from Poland, 2 from Anguilla, 1 from Andorra, 1 from Thailand), 638 with missing core data, and 97 without neonatal TSH screening. Ultimately, 12,593 infants were included in the study (Figure 1). The median GA was 35 weeks, the median BW was 2280 grams, and 6673 infants (52.99%) were male. The median age of mothers was 31.90 years, with most (12,062, 95.78%) identified as Han Chinese. Singleton pregnancies were predominant (53.70%), and 67.50% of the infants were conceived spontaneously (Table 1).

**Table 1:** Characteristics of study participants in different gestational age groups.

Criteria for classification	Overall preterm (<37 wk)	Late preterm (34∼<37 wk)	Moderate preterm (32∼<34 wk)	Very preterm (28∼<32wk)	Extremely preterm (<28 wk)
Gestational age, median(IQR), wk	35 (33–36)	36 (35–36)	33 (32–33)	30 (29–31)	27 (26–27)
Birth weight, median (IQR), g	2,280 (1,880–2,590)	2,430 (2,190–2,680)	1,840 (1,630–2,030)	1,380.00 (1,200–1,590)	950.00 (835–1,050)
Maternal age, median (IQR), year	31.90 (29.20–34.80)	32.00 (29.40–35.00)	31.40 (28.40–34.40)	31.50 (28.30–34.20)	31.30 (29.60–34.30)
Sex, *n* (%)					
Male	6,673 (52.99)	4,907 (52.82)	924 (53.38)	703 (52.42)	139 (60.17)
Female	5,920 (47.01)	4,383 (47.18)	807 (46.62)	638 (47.58)	92 (40.83)
Ethnicity, *n* (%)					
Han	12,062 (95.78)	8,962 (96.47)	1,639 (94.69)	1,245 (92.84)	216 (93.51)
Others	531 (4.22)	328 (3.53)	92 (5.31)	96 (7.16)	15 (6.49)
Gestation type, *n* (%)					
Singleton	6,762 (53.70)	5,062 (54.49)	815 (47.08)	752 (56.08)	133 (57.58)
Multiple	5,831 (46.30)	4,228 (45.51)	916 (52.92)	589 (43.92)	98 (42.42)
Conception mode, *n* (%)					
Spontaneous conception	8,500 (67.50)	6,285 (67.65)	1,178 (68.05)	911 (67.93)	126 (54.55)
*In vitro* fertilization (IVF)	4,093 (32.50)	3,005 (32.35)	553 (31.95)	430 (32.07)	105 (45.45)

### TSH and GA

Among all 12,593 preterm infants, the average screening time was 5.11 ± 3.08 days, and TSH levels ranged from 0.10 to 5.70 μIU/mL. The preterm infants were divided into four groups based on GA: late preterm (LP, 34∼<37 weeks, *n* = 9,290), moderate preterm (MP, 32∼<34 weeks, *n* = 1,731), very preterm (VP, 28∼<32 weeks, *n* = 1,341), and extremely preterm (EP, <28 weeks, *n* = 231). The TSH reference ranges for each group were 0.20–5.60 μIU/mL, 0.10–5.75 μIU/mL, 0.10–6.60 μIU/mL, and 0.10–5.21 μIU/mL, respectively (Table 2). According to the results of the multivariate generalized linear model, for every one-week increase in GA, the TSH levels increased by 1% [relative risk [RR] = 1.01, 95% confidence interval [CI]: 1.00–1.03, *P* < 0.05] (Table 3).

**Table 2:** Percentiles of TSH values (μIU/mL) for preterm infants.

Criteria for classification	*n*	Time of specimen collection, mean (SD), day	TSH value, μIU/mL (95% CI)						
			2.5th (95% CI)	10th (95% CI)	25th (95% CI)	50th (median)	75th (95% CI)	90th (95% CI)	97.5th (95% CI)
Overall	12,593	5.11 (3.08)	0.10 (0.10–0.20)	0.40 (0.40–0.40)	0.70 (0.7–0.8)	1.30 (1.3–1.4)	2.30 (2.2–2.3)	3.60 (3.50–3.63)	5.70 (5.52–5.86)
Gestational age									
Late preterm (34∼<37 wk)	9,290	4.98 (2.79)	0.20 (0.20–0.20)	0.40 (0.40–0.40)	0.80 (0.70–0.80)	1.40 (1.30–1.40)	2.30 (2.20–2.30)	3.50 (3.40–3.60)	5.60 (5.30–5.78)
Moderate preterm (32∼<34 wk)	1,731	4.56 (1.92)	0.10 (0.10–0.10)	0.30 (0.30–0.40)	0.70 (0.60–0.70)	1.30 (1.20–1.40)	2.30 (2.20–2.40)	3.60 (3.40–3.76)	5.75 (5.26–6.40)
Very preterm (28∼<32wk)	1,341	5.90 (4.18)	0.10 (0.10–0.10)	0.30 (0.30–0.30)	0.60 (0.60–0.70)	1.30 (1.20–1.30)	2.30 (2.20–2.40)	3.80 (3.40- 4.10)	6.60 (6.0–7.10)
Extremely preterm (<28 wk)	231	9.56 (7.05)	0.10 (0.10–0.10)	0.20 (0.10–0.30)	0.60 (0.40–0.70)	1.10 (0.90–1.30)	1.80 (1.60–2.20)	3.50 (2.60–3.70)	5.21 (4.20–6.10)
Age-appropriate									
Small for gestational age	1,902	5.06 (3.18)	0.10 (0.10–0.20)	0.41 (0.40–0.50)	0.80 (0.80–0.90)	1.50 (1.50–1.60)	2.60 (2.50–2.70)	4.10 (3.90–4.40)	6.70 (6.30–7.10)
Appropriate for gestational age	10,691	5.12 (3.06)	0.14 (0.10–0.20)	0.40 (0.40–0.40)	0.70 (0.70–0.70)	1.30 (1.30–1.30)	2.20 (2.20–2.24)	3.40 (3.37–3.50)	5.50 (5.30–5.70)
Birth weight									
Normal birth weight (≥2,500 g)	4,005	5.14 (2.92)	0.20 (0.10–0.20)	0.40 (0.40–0.40)	0.80 (0.70–0.80)	1.32 (1.30–1.40)	2.20 (2.20–2.30)	3.40 (3.30–3.40)	5.28 (5.00–5.42)
Low birth weight (1,500∼<2,500 g)	7,124	4.79 (2.52)	0.20 (0.10–0.20)	0.40 (0.40–0.40)	0.70 (0.70–0.80)	1.30 (1.30–1.40)	2.20 (2.20–2.30)	3.60 (3.40–3.70)	5.70 (5.57–6.00)
Very low birth weight (1,000∼<1,500 g)	1,208	6.00 (4.28)	0.10 (0.10–0.10)	0.30 (0.30–0.40)	0.70 (0.60–0.80)	1.40 (1.30–1.50)	2.42 (2.30–2.60)	3.87 (3.60–4.20)	6.59 (5.88–6.80)
Extremely low birth weight (<1,000 g)	256	9.24 (6.89)	0.10 (0.01–0.10)	0.30 (0.20–0.35)	0.60 (0.50–0.78)	1.30 (1.20–1.61)	2.40 (2.20–3.01)	4.45 (3.61–5.20)	7.36 (6.00–8.55)

**Table 3:** Analysis of influencing factors on thyroid-stimulating hormone levels in preterm infants.

Criteria for classification	Univariate analysis		Multivariate analysis	
	RR(95%CI)	*P* value	aRR(95%CI)	*P* value
Gestational age	1.01 (0.99–1.01)	*P* = 0.07	1.01 (1.00–1.03)	*P* < 0.05
Birth weight	0.99 (0.99–0.99)	*P* < 0.05	0.99 (0.99–1.00)	*P* = 0.09
Maternal age	0.99 (0.99–0.99)	*P* < 0.05	0.99 (0.99–1.00)	*P* = 0.06
Sex				
Male	ref		Ref	
Female	1.02 (0.99–1.05)	*P* = 0.08	1.00 (0.98–1.04)	*P* = 0.48
Ethnic				
Han	Ref		Ref	
Others	0.99 (0.92–1.05)	*P* = 0.72	0.97 (0.91–1.04)	*P* = 0.42
Age-appropriate				
Small for gestational age	1.18 (1.14–1.23)	*P* < 0.05	1.15 (1.10–1.21)	*P* < 0.05
Appropriate for gestational age	ref		Ref	
Gestation				
Singleton	ref		Ref	
Multiple	0.99 (0.96–1.02)	*P* = 0.44	0.97 (0.94–1.01)	*P* = 0.10
Pregnancy				
Spontaneous	ref		Ref	
IVF	0.97 (0.94–0.99)	*P* < 0.05	0.98 (0.94–1.01)	*P* = 0.14

### TSH and BW

According to BW, the TSH reference ranges were NBW (0.20–5.28 μIU/mL), LBW (0.20–5.70 μIU/mL), VLBW (0.10–6.59 μIU/mL), and ELBW (0.10–7.36 μIU/mL). According to the results of the univariate generalized linear model, higher BW was associated with lower TSH levels (RR = 0.99, 95%CI:0.99–0.99, *P* < 0.05). However, this association was not observed in the adjusted generalized linear model (Table 3).

### TSH and SGA

We further categorized all the preterm infants into two subgroups: SGA (*n* = 1,902) and AGA (*n* = 10,691). The TSH reference ranges for each group were (0.10–6.70 μIU/mL) and (0.14–5.50 μIU/mL). The median TSH in SGA was higher than in AGA, and the difference was statistically significant. According to the results of the multivariate generalized linear model, compared with AGA (appropriate for GA), the TSH level of SGA was higher (RR = 1.15, 95% CI: 1.10–1.21, *P* < 0.05) (Table 3).

### TSH and PNA

**Figure 2:**
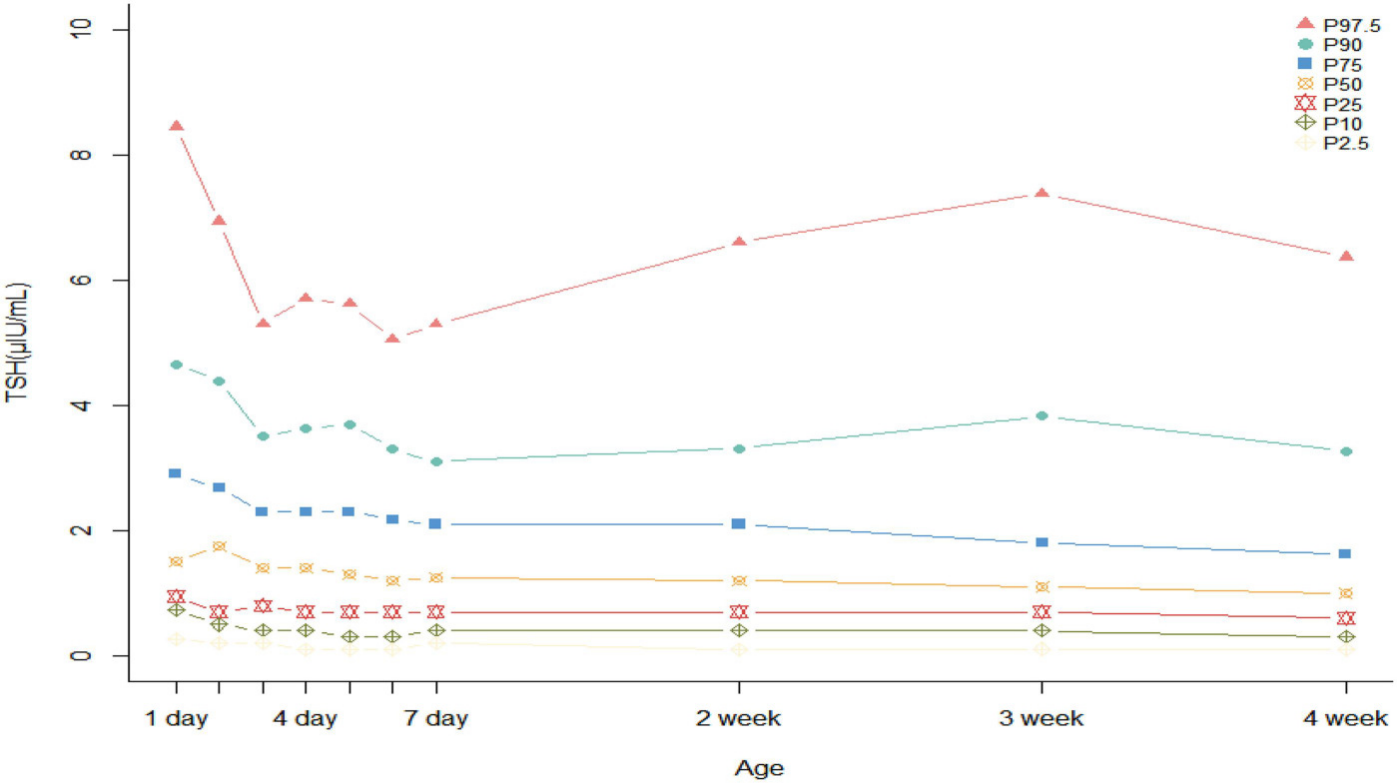
Plots of the 2.5th, 10th, 25th, 50th, 75th, 90th, and 97.5th TSH percentiles in preterm infants from the first to fourth week of life.

To study the dynamic changes of TSH in preterm infants, we calculated the TSH reference intervals separately for the first through fourth weeks after birth. Newborn screening is typically finished within the first week post-birth. To better monitor the changes in TSH, we computed the TSH values for each day of the first week, from day 1 to day 7 after birth. Because of the limitations in sample size, the TSH values for the second to fourth weeks were calculated on a weekly basis. The results indicated that the TSH cutoff value (97th percentile) peaked at 24 h after birth, then declined gradually within 48 h, and stabilized after 72 h (Figure 2). The reference intervals for TSH were as follows: first week (*n* = 11,312, 0.12–5.60 μIU/mL), second week (*n* = 866, 0.10–6.60 μIU/mL), third week (*n* = 331, 0.11–7.38 μIU/mL), fourth week (*n* = 84, 0.10–6.37 μIU/mL) (Table 4). The results showed that the weekly calculated TSH critical values first increased and then decreased, with a maximum of 7.38 μIU/mL in the third postnatal week. However, within the first week, as the time of blood collection passed, the TSH values across all percentiles decreased.

**Table 4:** Percentiles of TSH values (μIU/mL) by the time of specimen collection for overall preterm infants.

Postnatal age, week/day	*n*	TSH value, μIU/mL (95% CI)						
		2.5th	10th	25th	50th (Median)	75th	90th	97.5th
Week 1	11,312	0.12 (0.10–0.20)	0.40 (0.40–0.40)	0.70 (0.70–0.80)	1.40 (1.30–1.40)	2.30 (2.26–2.30)	3.60 (3.50–3.70)	5.60 (5.40–5.80)
Day 1	18	0.26 (0.00–0.90)	0.74 (0.72–0.81)	0.95 (0.65–1.45)	1.50 (0.75–2.20)	2.90 (1.45–3.70)	4.66 (2.60–9.60)	8.45 (3.34–9.60)
Day 2	62	0.21 (0.1–0.50)	0.50 (0.31–0.60)	0.70 (0.60–1.20)	1.75 (1.20–2.10)	2.68 (2.18–3.27)	4.39 (2.99–6.59)	6.94 (4.50–8.40)
Day 3	3,148	0.20 (0.20–0.20)	0.40 (0.40–0.50)	0.80 (0.80–0.80)	1.40 (1.30–1.40)	2.30 (2.20–2.35)	3.50 (3.30–3.50)	5.30 (5.10–5.65)
Day 4	5,868	0.10 (0.10–0.20)	0.40 (0.40–0.40)	0.70 (0.70–0.80)	1.40 (1.30–1.40)	2.30 (2.30–2.40)	3.63 (3.50–3.78)	5.71 (5.53–5.93)
Day 5	1,337	0.10 (0.10–0.10)	0.30 (0.30–0.40)	0.70 (0.60–0.70)	1.30 (1.20–1.40)	2.30 (2.14–2.40)	3.70 (3.4–3.94)	5.62 (5.10–6.18)
Day 6	593	0.10 (0.10–0.20)	0.30 (0.30–0.40)	0.70 (0.60–0.70)	1.20 (1.10–1.30)	2.18 (1.90–2.30)	3.30 (3.10–3.60)	5.05 (4.52–6.00)
Day 7	286	0.20 (0.10–0.30)	0.40 (0.30–0.50)	0.70 (0.62–0.80)	1.25 (1.10–1.40)	2.10 (1.90–2.30)	3.10 (2.70–3.50)	5.29 (3.90–6.10)
Week 2	866	0.10 (0.10–0.20)	0.40 (0.31–0.41)	0.70 (0.60–0.74)	1.20 (1.10–1.30)	2.10 (1.90–2.20)	3.30 (3.0–3.70)	6.60 (5.00–8.00)
Week 3	331	0.11 (0.10–0.24)	0.40 (0.30–0.40)	0.70 (0.60–0.77)	1.10 (1.00–1.22)	1.80 (1.60–2.20)	3.83 (2.60–5.06)	7.38 (6.12–9.00)
Week 4	84	0.10 (0.08–0.21)	0.30 (0.20–0.40)	0.60 (0.40–0.70)	1.00 (0.80–1.25)	1.62 (1.40–2.12)	3.27 (2.10–6.00)	6.37 (3.65–11.33)

In this study, we calculated the TSH reference values for each group at various blood collection times after stratifying the participants based on GA, BW, and their SGA status (as presented in the Supplementary Tables). Median TSH values across different blood collection times were plotted as line graphs for each group (Supplementary Figure S1). Among the LP, MP, and VP groups, a similar pattern was observed. Median TSH peaked in the second week after birth and then declined. The EP group, however, reached its peak in the third week. For the SGA and AGA groups, the most significant difference in TSH levels was observed during the first two weeks after birth, with the disparity gradually lessening in the third and fourth weeks. In the NBW, LBW, and VLBW groups, we observed a similar trend in which TSH levels decreased significantly with increasing PNA. In contrast, the ELBW group peaked in the third week and then dropped.

## Discussion

This retrospective study, involving 12,593 neonates with GA less than 37 weeks, constitutes one of the largest samples in the study of TSH levels in preterm infants. The results revealed a TSH cutoff value of 5.70 μIU/mL for preterm infants, lower than the normal term reference value, and there were corresponding results for different BW and different GA. We analyzed the effects of GA, BW, and SGA on TSH in preterm infants separately; these data help to fill the knowledge gap in understanding postnatal TSH levels in Chinese preterm infants.

The results showed that the TSH cutoff value for preterm infants was 5.70 IU/mL, which is smaller than the current cut-off value in our laboratory (9.00 μIU/mL) and is lower than most previous studies. Although the cutoff values observed in this study were significantly lower than those reported in previous studies from Lombardy, Italy (2009) (18) and Greece (2010) (19), this finding was consistent with the recent global trend of lowering the TSH cutoff values in the NBST (3). This may be partly attributed to the improvement in the nutritional status of pregnant and lactating women in recent years, especially the supplementation of iodine (20, 21). It could also be attributed to the differences in ethnicity and region. Although the reduction in the threshold will increase the false positive rate of congenital hypothyroidism (CH) and the screening cost (22), there is a wealth of strong evidence showing (23) that untreated permanent CH can lead to significant negative neurodevelopmental outcomes. Therefore, it is essential to reduce the rate of missed diagnosis of CH.

We analyzed the effects of GA, BW, and SGA on TSH in preterm infants separately. According to our research results, there are differences in the cut-off values of TSH among different GA groups and BW groups. Compared to preterm AGA infants, SGA infants had higher serum TSH levels, consistent with the findings of Gerdi Tuli et al. (24) I. F. González, F. Grob, et al. (25, 26). However, Xin Lin, O. N. Aktas, F. C. Kilchemmann, et al., suggested that GA had no significant effect on TSH (11, 27, 28). A possible explanation for this discrepancy is that the HPT axis of preterm infants matures gradually in the third trimester of pregnancy (30–35 weeks of gestation) and is not fully mature until the early neonatal period (13). This important finding can provide strong evidence to support the definition of TSH cut-off values for preterm infants with different GA and BW.

We also investigated the changes in the values of TSH due to PNA. During the first week after birth, the 97.5th percentile value of TSH was the highest on the first day, and then it steadily decreased until it was close to the cut-off value of TSH for preterm infants (5.70 μIU/mL) on the fourth day, and then showed a slight upward trend afterwards. In contrast, when examining weeks 1–4 overall, TSH tended to increase first and then decrease, reaching a peak of 7.38 μIU/mL in the third postnatal week and then decreasing gradually in the fourth week, approaching the value measured in the first week, exhibiting a phenomenon of delayed elevation of thyroid-stimulating hormone.

In the Supplementary Materials, we analyzed how TSH reference ranges for preterm infants across different GA groups varied with PNA (Supplementary Tables 1–S4). We found that TSH cutoff values peaked in the third week for late preterm, very preterm, and extremely preterm groups (5.73 μIU/mL, 8.72 μIU/mL, and 5.86 μIU/mL, respectively). This trend largely aligns with the overall pattern of TSH cutoff values across all preterm infants. Only the moderate preterm group exhibited a peak TSH cutoff value at week 4 postnatal age. This discrepancy may be attributed to small sample sizes in this group at 3 and 4 weeks postnatal age, which may have introduced statistical bias. Future studies should expand sample sizes to address this observation. These findings suggest that TSH changes dynamically with PNA. Consistent with the findings of Dinushan C. Kaluarachchi et al. Kilberg et al. studied the range of reference values of TSH in preterm infants of different GA from day 1 to day 14 of life. It was concluded that the TSH values may be different for different PNAs (29–31). The above findings suggest that appropriate TSH cutoff values should be established for different PNAs in preterm infants. Meanwhile, TSH was affected by SGA and differed significantly from AGA in the first two weeks of postnatal life, suggesting that TSH reference intervals suitable for SGA children can be established in different GA groups within 2 weeks of birth.

This study is one of the largest studies, filling the gap regarding preterm infants in western China for different GA and different BW. The reference range is lower than the normal term cut-off value (9.0 IU/mL), and different from other studies, may be suitable for West Regional China. In our study, TSH levels are affected by GA and PNA, and exhibit different trends in different GA groups, suggesting the importance of continuous monitoring of thyroid function in preterm infants (especially GA <32 weeks). Therefore, there is a need to establish optimal time points for newborn screening for different GA of preterm infants, and there is a need to establish different TSH cut-off values at different screening time points. For example, We further analyzed the TSH values of preterm infants of different GA at different time points of blood collection (Supplementary Tables 1–S4), and found that the 50th and 97.5th TSH values of preterm infants <28 weeks of age were significantly lower than those of the other groups during the first postnatal week, suggesting that it may be necessary to establish a lower cut-off value of TSH in extreme-preterm infants to avoid an increased rate of false negatives.

Our study also has some limitations; Firstly, as the data originated from our hospital, this is a single-center study, and the sample size of the extremely preterm group is rather small. To assess the stability of the results, we calculated 95% confidence intervals for each percentile. The results showed relatively wide confidence intervals for birth days 1 and 2, indicating that, due to the smaller sample size during this period, the precision and population representativeness of the percentile results were limited. Diverse population characteristics and sample sizes across studies may lead to varying outcomes. Secondly, NBST employs TRFIA using whole blood dried blood spots. While TSH demonstrates good stability at high concentrations ranges within this assay, its detection accuracy is limited in low concentration ranges. Consequently, establishing sufficiently precise lower reference limits proves challenging. However, our core objective in calculating TSH reference ranges is to identify the critical threshold (the 97.5th percentile) to provide a reference for clinical disease screening. Thus, the lower percentile reference range is descriptive only and lacks clinical diagnostic significance. Moreover, preterm infants often exhibit fluctuations in hematocrit, and whole blood TSH measurements can be affected by blood dilution/concentration, potentially leading to systematic bias in TSH values. Furthermore, some patients are discharged before the second week of life, prohibiting delayed follow-up of all babies, which may result in missed visits and impaired data inclusion. Additionally, we eliminated preterm infants treated with levothyroxine following the diagnosis of THOP and transient hyperthyrotropinemia, but the TSH criteria for diagnosis and treatment vary by laboratory, and it is unknown how these factors may affect long-term neurodevelopmental results. So far, there have been fewer clinical trials or prospective studies of levothyroxine replacement therapy, and the evidence in the available literature is insufficient to evaluate whether TH medication reduces the prevalence of neurological sequelae in these patients (31–37). As an increasing number of preterm infants are now being diagnosed with these conditions, removing these newborns may impact the representativeness of the population and bias the study's conclusions. The effects of these factors on thyroid function in preterm newborns should be further investigated in the upcoming investigations.

There is no global consensus on screening for CH in preterm newborns, and screening procedures vary by country. On the one hand, due to the high number of postnatal complications in preterm infants, there are no criteria for defining healthy preterm infants, and the inclusion and exclusion criteria for the data are variable. On the other hand, preterm infants’ thyroid axis is still developing, their TSH fluctuates and is unable to set the same benchmark as term infants. Cutoff values for TSH that apply to preterm infants with varying GA and PNA must be established by multicenter, large-scale cohort studies. Therefore, standardized screening guidelines should be created to ensure consistent CH management and treatment.

## Conclusion

The results of this study revealed that TSH cut-off values in preterm infants are affected by GA, BW, and PNA, and TSH levels were significantly higher in SGA infants than in AGA infants. We established TSH cut-off values suitable for different conditions of preterm infants, providing a scientific basis for establishing GA-, BW-, and PNA-specific TSH screening cutoffs for preterm infants, and this is of great value for the standardized diagnosis and management of CH.

## Data Availability

The datasets presented in this article are not readily available because the raw data supporting the conclusions of this article contain information that compromises patient privacy and confidentiality. Therefore, data are not publicly available, but can be made available from the authors upon ethical approval. Requests to access the datasets should be directed to the corresponding author.
