# Supplementary material for: Exploring reference ranges for thyroid-stimulating hormone in neonatal screening tests for preterm infants: a 5-year retrospective study
Source: Front Pediatr. 2026 Apr 8;14:1781279. doi: 10.3389/fped.2026.1781279 (PMC13099535; doi:10.3389/fped.2026.1781279)
Supplement: Supplementary file 1 [file Supplementaryfile1.docx]

**Supplementary** **Table 1 Percentiles of TSH values (μIU/mL) by the time of specimen collection for late preterm (34~<37 wk) infants**

| Age, week/day | n | TSH value, μIU/mL (95% CI) | | | | | | |
| --- | --- | --- | --- | --- | --- | --- | --- | --- |
|  |  | 2.5th | 10th | 25th | 50th (Median) | 75th | 90th | 97.5th |
| Week 1 | 8416 | 0.20(0.20-0.20) | 0.40(0.40-0.44) | 0.80(0.80-0.80) | 1.40(1.40-1.40) | 2.30(2.30-2.36) | 3.60(3.50-3.70) | 5.60(5.30-5.80) |
| 1 | 8 | 0.65(0.60-1.33) | 0.81(0.60-1.95) | 1.05(0.60-2.60) | 1.90(0.90-6.90) | 3.98(1.40-9.60) | 7.71(2.40-9.60) | 9.13(2.40-9.60) |
| 2 | 41 | 0.10(0.10-0.50) | 0.50(0.10-0.60) | 0.60(0.50-1.20) | 1.70(0.92-2.40) | 2.60(2.10-3.20) | 4.40(2.70-7.10) | 7.10(3.20-8.40) |
| 3 | 2450 | 0.20(0.20-0.20) | 0.50(0.48-0.50) | 0.80(0.80-0.90) | 1.50(1.40-1.50) | 2.30(2.20-2.40) | 3.50(3.31-3.70) | 5.38(5.10-5.70) |
| 4 | 4249 | 0.20(0.20-0.20) | 0.40(0.40-0.50) | 0.80(0.70-0.80) | 1.40(1.40-1.45) | 2.32(2.30-2.40) | 3.70(3.50-3.80) | 5.70(5.40-5.90) |
| 5 | 962 | 0.10(0.10-0.20) | 0.31(0.30-0.40) | 0.70(0.70-0.80) | 1.40(1.40-1.50) | 2.30(2.20-2.40) | 3.79(3.40-4.00) | 5.64(5.10-6.15) |
| 6 | 459 | 0.10(0.10-0.20) | 0.30(0.30-0.40) | 0.70(0.60-0.80) | 1.30(1.20-1.40) | 2.20(2.00-2.30) | 3.32(3.10-3.80) | 4.96(4.50-5.91) |
| 7 | 247 | 0.20(0.10-0.30) | 0.40(0.30-0.50) | 0.70(0.60-0.80) | 1.20(1.10-1.40) | 2.05(1.85-2.30) | 3.10(2.84-3.56) | 5.30(3.84-6.37) |
| Week 2 | 614 | 0.10(0.10-0.20) | 0.40(0.30-0.40) | 0.65(0.60-0.70) | 1.10(1.00-1.20) | 1.80(1.70-2.00) | 2.90(3.60-3.14) | 5.31(4.43-7.2) |
| Week 3 | 231 | 0.12(0.06-0.28) | 0.40(0.30-0.40) | 0.62(0.55-0.70) | 1.00(0.90-1.10) | 1.50(1.40-1.70) | 2.50(2.10-3.40) | 5.73(3.83-36.6) |
| Week 4 | 29 | 0.23(0.08-0.40) | 0.38(0.26-0.58) | 0.60(0.40-0.70) | 0.70(0.60-1.00) | 1.10(0.80-1.70) | 1.94(1.12-3.22) | 3.23(1.63-3.30) |

Abbreviation: TSH, thyroid-stimulating hormone.

**Supplementary Table 2 Percentiles of TSH values (μIU/mL) by the time of specimen collection for moderate preterm (32~<34 wk) infants**

| Age, week/day | n | TSH value, μIU/mL (95% CI) | | | | | | |
| --- | --- | --- | --- | --- | --- | --- | --- | --- |
|  |  | 2.5th | 10th | 25th | 50th (Median) | 75th | 90th | 97.5th |
| Week 1 | 1648 | 0.10(0.10-0.10) | 0.30(0.30-0.40) | 0.70(0.60-0.70) | 1.30(1.20-1.35) | 2.30(2.18-2.40) | 3.54(3.38-3.75) | 5.66(5.20-6.17) |
| 1 | 6 | 0.10(0.00-1.25) | 0.40(0.00-1.80) | 0.88(0.00-2.62) | 1.70(0.40-3.65) | 3.28(1.03-3.70) | 3.65(1.70-3.70) | 3.69(2.15-3.70) |
| 2 | 11 | 0.55(0.50-1.10) | 0.70(0.50-1.30) | 1.00(0.60-2.00) | 1.90(0.90-3.30) | 2.70(1.60-4.80) | 4.30(2.10-5.30) | 5.05(2.10-5.30) |
| 3 | 406 | 0.11(0.10-0.20) | 0.40(0.30-0.40) | 0.70(0.60-0.80) | 1.30(1.20-1.40) | 2.20(2.00-2.49) | 3.30(3.10-3.85) | 5.29(4.30-6.34) |
| 4 | 975 | 0.10(0.10-0.20) | 0.30(0.30-0.40) | 0.70(0.60-0.80) | 1.30(1.21-1.40) | 2.30(2.20-2.43) | 3.60(3.40-4.00) | 5.94(5.45-6.70) |
| 5 | 186 | 0.10(0.10-0.30) | 0.35(0.30-0.40) | 0.70(0.50-0.80) | 1.20(1.20-1.40) | 2.20(2.00-2.49) | 3.58(3.10-3.85) | 5.10(4.40-6.50) |
| 6 | 47 | 0.10(0.10-0.22) | 0.20(0.10-0.41) | 0.55(0.35-0.90) | 1.10(0.80-1.30) | 1.70(1.20-2.25) | 3.16(1.86-3.78) | 3.87(3.00-4.50) |
| 7 | 17 | 0.14(0.10-0.72) | 0.44(0.10-0.92) | 0.86(0.20-1.10) | 1.10(0.86-1.90) | 1.90(1.10-3.90) | 2.88(1.60-4.16) | 4.06(2.02-4.16) |
| Week 2 | 64 | 0.10(0.00-0.40) | 0.46(0.16-0.70) | 0.90(0.60-1.20) | 1.65(1.20-1.95) | 2.52(2.00-3.10) | 3.70(2.90-5.54) | 6.20(4.08-10.50) |
| Week 3 | 14 | 0.50(0.50-0.76) | 0.56(0.50-0.94) | 0.75(0.50-0.94) | 1.35(0.80-1.70) | 1.68(1.20-3.50) | 3.05(1.57-6.70) | 5.66(1.70-6.70) |
| Week 4 | 5 | 0.20(0.20-0.68) | 0.20(0.20-0.92) | 0.20(0.20-1.40) | 0.60(0.20-11.70) | 1.40(0.20-11.70) | 7.58(0.44-11.70) | 10.67(0.56-11.70) |

Abbreviation: TSH, thyroid-stimulating hormone.

**Supplementary** **Table 3 Percentiles of TSH values (μIU/mL) by the time of specimen collection for very preterm (28~<32wk) infants**

| Age, week/day | n | TSH value, μIU/mL (95% CI) | | | | | | |
| --- | --- | --- | --- | --- | --- | --- | --- | --- |
|  |  | 2.5th | 10th | 25th | 50th (Median) | 75th | 90th | 97.5th |
| Week 1 | 1119 | 0.10(0.10-0.10) | 0.30(0.20-0.30) | 0.60 (0.50-0.70) | 1.20(1.10-1.30) | 2.20 (2.10-2.30) | 3.42 (3.20-3.80) | 5.80(5.30-6.41) |
| 1 | 4 | 0.93(0.90-1.60) | 1.02(0.90-1.69) | 1.20(0.90-2.28) | 1.45(0.90-2.26) | 1.85(1.08-2.60) | 2.30(1.30-2.60) | 2.53(1.30-2.60) |
| 2 | 10 | 0.37(0.30-1.67) | 0.57(0.30-1.74) | 1.30(0.38-1.97) | 1.85(1.10-3.05) | 2.65(1.75-6.07) | 4.19(2.10-6.80) | 6.15(2.2-6.80) |
| 3 | 262 | 0.10(0.00-0.20) | 0.30(0.30-0.40) | 0.60(0.50-0.76) | 1.20(1.09-1.45) | 2.30(2.00-2.70) | 3.40(3.00-3.86) | 5.11(4.18-6.09) |
| 4 | 589 | 0.10(0.10-0.10) | 0.30(0.20-0.30) | 0.60(0.50-0.70) | 1.20(1.10-1.30) | 2.20(2.07-2.40) | 3.70(3.20-4.02) | 5.73(4.99-6.51) |
| 5 | 162 | 0.10(0.10-0.10) | 0.20 (0.10-0.30) | 0.50(0.40 -0.60) | 1.15(0.90-1.30) | 1.90(1.70-2.30) | 3.17 (2.50 -3.70) | 6.39 (3.70 -7.19) |
| 6 | 74 | 0.10(0.00-0.20) | 0.23(0.20-0.49) | 0.58 (0.40-0.80) | 1.04 (0.80-1.30) | 2.20(1.40-2.74) | 3.30(2.54-5.64) | 6.54(3.47-7.90) |
| 7 | 18 | 0.70 (0.70-0.88) | 0.77(0.70-1.10) | 1.03(0.72 ~ 1.70) | 1.70(1.05-2.35) | 2.38(1.70-2.50) | 2.50(2.26-3.90) | 3.31(2.40-3.90) |
| Week 2 | 138 | 0.30 (0.10-0.40) | 0.50 (0.37-0.70) | 1.00(0.70-1.20) | 1.65(1.40-1.90) | 2.70(2.40-3.30) | 4.93(3.80-6.80) | 8.23(6.67-20.30) |
| Week 3 | 57 | 0.20(0.20-0.44) | 0.46(0.20-0.80) | 1.00 (0.60-1.40) | 1.60(1.40-2.00) | 4.40(2.00-6.30) | 6.88 (4.98-8.30) | 8.72(7.02-11.80) |
| Week 4 | 27 | 0.17(0.10-0.40) | 0.36(0.16-0.60) | 0.60 (0.40-1.15) | 1.30(0.80-1.90) | 2.00(1.50-4.05) | 4.38(1.98-6.56) | 6.54(3.17-6.80) |

Abbreviation: TSH, thyroid-stimulating hormone.

**Supplementary** **Table 4 Percentiles of TSH values (μIU/mL) by the time of specimen collection for extremely preterm (<28 wk) infants**

| Age, week/day | n | TSH value, μIU/mL (95% CI) | | | | | | |
| --- | --- | --- | --- | --- | --- | --- | --- | --- |
|  |  | 2.5th | 10th | 25th | 50th (Median) | 75th | 90th | 97.5th |
| Week 1 | 129 | 0.10 (0.00-0.10) | 0.20(0.10-0.30) | 0.40(0.30-0.60) | 0.90(0.70-1.20) | 1.50(1.40-1.80) | 2.42(2.04-3.61) | 5.20(3.36-6.10) |
| 3 | 30 | 0.10 (0.10-0.20) | 0.19 (0.10-0.40) | 0.43(0.20-0.70) | 0.70(0.55-0.90) | 1.25(0.78-1.82) | 1.91(1.30-3.33) | 3.38(1.68-3.61) |
| 4 | 55 | 0.04(0.00-0.23) | 0.24(0.10-0.40) | 0.45(0.30-0.70) | 0.90(0.70-1.20) | 1.55(1.20-2.30) | 3.10(1.76-5.66) | 5.95(3.11-9.60) |
| 5 | 27 | 0.10(0.10-0.23) | 0.10(0.10-0.36) | 0.35(0.10-0.80) | 1.10(0.40-1.50) | 1.60(1.15-2.50) | 3.41(1.58-5.10) | 5.09(2.43-5.25) |
| 6 | 13 | 0.45(0.30-1.20) | 0.82(0.30-1.32) | 1.20(0.80-1.60) | 1.40(1.20-1.80) | 1.80(1.40-2.20) | 2.20(1.76-2.20) | 2.20(1.80-2.20) |
| 7 | 4 | 0.32(0.30-1.40) | 0.39(0.30-1.40) | 0.53(0.30-1.40) | 1.00(0.30-1.50) | 1.43(0.58-1.50) | 1.47(0.60-1.50) | 1.49(0.60-1.50) |
| Week 2 | 50 | 0.12 (0.10-0.44) | 0.41 (0.19-0.60) | 0.80(0.60-1.10) | 1.30(1.00-1.80) | 2.20(1.73-3.48) | 3.51(2.60-3.74) | 4.01(3.50-4.80) |
| Week 3 | 29 | 0.10(0.10-0.31) | 0.26(0.10-0.60) | 0.60 (0.3-1.4) | 1.50(0.90-2.10) | 2.90(1.80-4.00) | 4.04(2.84-5.64) | 5.86(3.73-7.40) |
| Week 4 | 23 | 0.10(0.10-0.43) | 0.30(0.10-0.84) | 0.75(0.30-1.20) | 1.20 (1.00-1.50) | 1.64(1.40-3.20) | 3.42(1.58-5.64) | 5.01(2.24-6.00) |

Abbreviation: TSH, thyroid-stimulating hormone.

**Supplementary** **Table 5 Percentiles of TSH values (μIU/mL) by the time of specimen collection for SGA infants**

| Age, week/day | n | TSH value, μIU/mL (95% CI) | | | | | | |
| --- | --- | --- | --- | --- | --- | --- | --- | --- |
|  |  | 2.5th | 10th | 25th | 50th (Median) | 75th | 90th | 97.5th |
| Week 1 | 1740 | 0.10(0.10-0.20) | 0.40(0.40-0.50) | 0.80(0.80-0.90) | 1.50(1.50-1.60) | 2.60(2.42-2.70) | 4.04(3.80-4.21) | 6.50(6.05-6.80) |
| 1 | 4 | 0.64(0.60-2.30) | 0.75(0.60-2.30) | 0.98(0.60-2.30) | 1.70(0.60-2.60) | 2.38(1.02-2.60) | 2.51(1.10-2.60) | 2.59(1.10-2.60) |
| 2 | 6 | 0.60(0.60-1.38) | 0.60(0.60-1.60) | 0.78(0.60-1.95) | 1.60(0.60-2.70) | 2.05(1.12-3.30) | 2.70(1.60-3.30) | 3.15(1.82-3.30) |
| 3 | 448 | 0.30(0.10-0.32) | 0.54(0.50-0.60) | 0.90(0.80-1.00) | 1.60(1.40-1.70) | 2.50(2.30-2.70) | 3.60(3.33-4.27) | 6.20(5.30-7.00) |
| 4 | 970 | 0.12(0.10-0.20) | 0.40(0.40-0.50) | 0.80(0.80-0.90) | 1.57(1.50-1.60) | 2.70(2.50-2.90) | 4.40(4.04-4.62) | 6.70(6.14-7.28) |
| 5 | 198 | 0.10(0.09-0.20) | 0.30(0.20-0.45) | 0.70(0.60-0.90) | 1.30(1.10-1.46) | 2.40(1.90-2.80) | 3.63(3.20-4.29) | 5.71(4.50-6.42) |
| 6 | 89 | 0.10(0.04-0.30) | 0.38(0.20-0.48) | 0.70(0.50-1.00) | 1.50(1.11-1.80) | 2.30(2.20-3.30) | 3.60(3.00-4.20) | 5.76(3.68-7.10) |
| 7 | 25 | 0.28(0.10-0.70) | 0.50(0.22-1.04) | 1.00(0.50-1.40) | 1.60(1.10-2.50) | 3.10(1.60-3.60) | 3.72(2.86-3.90) | 3.90(3.42-3.90) |
| Week 2 | 99 | 0.10(0.05-0.35) | 0.48(0.20-0.60) | 0.89(0.60-1.15) | 1.80(1.40-2.20) | 3.00(2.50-3.46) | 4.92(3.44-7.60) | 10.79(6.43-20.96) |
| Week 3 | 45 | 0.40(0.40-0.70) | 0.64(0.40-0.79) | 0.80(0.70-1.10) | 1.30(1.10-2.40) | 2.90(2.40-5.20) | 5.32(3.34-7.22) | 7.28(5.20-36.6) |
| Week 4 | 18 | 0.39(0.30-0.64) | 0.57(0.30-0.70) | 0.70(0.52-1.00) | 1.00(0.70-1.60) | 1.78(1.00-5.35) | 3.46(1.40-11.7) | 9.45(2.01-11.7) |

Abbreviation: TSH, thyroid-stimulating hormone.

**Supplementary** **Table 6 Percentiles of TSH values (μIU/mL) by the time of specimen collection for AGA infants**

| Age, week/day | n | TSH value, μIU/mL (95% CI) | | | | | | |
| --- | --- | --- | --- | --- | --- | --- | --- | --- |
|  |  | 2.5th | 10th | 25th | 50th (Median) | 75th | 90th | 97.5th |
| Week 1 | 9572 | 0.13(0.10-0.20) | 0.40(0.40-0.40) | 0.70(0.70-0.77) | 1.30(1.30-1.40) | 2.20(2.20-2.30) | 3.50(3.40-3.60) | 5.40(5.30-5.60) |
| 1 | 14 | 0.26(0.00-0.97) | 0.83(0.00-1.16) | 0.95(0.8-1.6) | 1.50(1.00-3.35) | 3.45(1.40-6.90) | 5.94(2.58-9.60) | 8.72(3.47-9.60) |
| 2 | 56 | 0.18(0.10-0.50) | 0.50(0.30-0.65) | 0.70(0.58-1.20) | 1.75(1.20-2.25) | 2.73(2.22-3.90) | 4.50(3.00-6.80) | 6.98(4.53-8.40) |
| 3 | 2700 | 0.20(0.19-0.2) | 0.40(0.40-0.50) | 0.80(0.80-0.80) | 1.40(1.31-1.43) | 2.20(2.16-2.30) | 3.40(3.20-3.60) | 5.20(4.97-5.60) |
| 4 | 4898 | 0.13(0.10-0.20) | 0.40(0.40-0.40) | 0.70(0.70-0.78) | 1.30(1.30-1.40) | 2.30(2.20-2.30) | 3.50(3.39-3.60) | 5.50(5.30-5.70) |
| 5 | 1139 | 0.10(0.10-0.10) | 0.30(0.30-0.40) | 0.70(0.60-0.70) | 1.30(1.20-1.40) | 2.30(2.10-2.35) | 3.70(3.30-4.00) | 5.51(5.10-6.18) |
| 6 | 504 | 0.10(0.10-0.20) | 0.30(0.23-0.40) | 0.70(0.60-0.70) | 1.20(1.10-1.30) | 2.10(1.80-2.20) | 3.20(2.90-3.57) | 5.00(4.54-5.80) |
| 7 | 261 | 0.20(0.10-0.30) | 0.40(0.30-0.50) | 0.70(0.60-0.80) | 1.20(1.10-1.40) | 2.00(1.80-2.20) | 3.00(2.50-3.40) | 5.30(3.90-6.29) |
| Week 2 | 767 | 0.10(0.10-0.20) | 0.40(0.30-0.40) | 0.70(0.60-0.70) | 1.14(1.10-1.20) | 1.96(1.80-2.10) | 3.00(2.74-3.44) | 5.67(4.79-7.04) |
| Week 3 | 286 | 0.10(0.10-0.20) | 0.32(0.30-0.40) | 0.61(0.53-0.70) | 1.10(10-1.20) | 1.70(1.50-1.90) | 3.30(2.30-4.40) | 7.39(5.32-8.91) |
| Week 4 | 66 | 0.10(0.08-0.20) | 0.25(0.10-0.40) | 0.43(0.32-0.80) | 1.00(0.80-1.30) | 1.60(1.37-2.25) | 3.25(1.90-4.85) | 6.00(3.30-6.80) |

Abbreviation: TSH, thyroid-stimulating hormone.

**Supplementary** **Table 7 Percentiles of TSH values (μIU/mL) by the time of specimen collection for normal birth weight (≥2500g) infants**

| Age, week/day | n | TSH value, μIU/mL (95% CI) | | | | | | |
| --- | --- | --- | --- | --- | --- | --- | --- | --- |
|  |  | 2.5th | 10th | 25th | 50th (Median) | 75th | 90th | 97.5th |
| Week 1 | 3546 | 0.20 (0.12-0.20) | 0.40(0.40-0.49) | 0.80(0.77-0.80) | 1.40(1.40-1.40) | 2.30(2.20-2.34) | 3.50(3.37-3.62) | 5.30(5.00-5.54) |
| 1 | 4 | 1.48(1.40-6.90) | 1.70(1.40-6.90) | 2.15(1.40-7.80) | 4.65 (1.40-9.60) | 7.58(2.40-9.60) | 8.79 (2.40-9.60) | 9.40(2.40-9.60) |
| 2 | 14 | 0.40(0.40-0.60) | 0.43(0.40-0.60) | 0.60(0.40-0.90) | 0.80(0.60-2.15) | 2.33(0.70-3.00) | 2.88(1.71-8.40) | 6.65(2.32-8.40) |
| 3 | 1071 | 0.20(0.13-0.30) | 0.50(0.40-0.60) | 0.90(0.80-0.90) | 1.42(1.40-1.50) | 2.30 (2.10-2.40) | 3.30(3.10-3.67) | 5.13(4.70-5.60) |
| 4 | 1656 | 0.20(0.14-0.20) | 0.40(0.40-0.50) | 0.80(0.70-0.80) | 1.40 (1.30-1.50) | 2.30(2.20-2.40) | 3.44(3.25-3.60) | 4.96 (4.60-5.30) |
| 5 | 437 | 0.10(0.10-0.20) | 0.34 (0.30-0.40) | 0.70(0.60-0.80) | 1.40(1.30-1.60) | 2.40(2.20-2.67) | 4.00(3.56-4.30) | 6.11(5.40-7.25) |
| 6 | 221 | 0.20(0.10-0.20) | 0.30(0.20-0.40) | 0.70(0.60-0.90) | 1.30(1.20-1.50) | 2.30(2.10-2.70) | 3.60(3.10-4.50) | 5.11 (4.65-6.00) |
| 7 | 143 | 0.16(0.10-0.30) | 0.40(0.30-0.50) | 0.70(0.60-0.80) | 1.10(10-1.30) | 1.80(1.60-2.35) | 3.00(2.50-3.75) | 5.25(3.60-6.40) |
| Week 2 | 325 | 0.10(0.10-0.20) | 0.37(0.30-0.42) | 0.70(0.60-0.78) | 1.10(1.00-1.20) | 1.80(1.70-2.14) | 2.70(2.46-3.00) | 4.57(3.89-5.50) |
| Week 3 | 125 | 0.10(0.05-0.26) | 0.30 (0.26-0.40) | 0.60(0.49-0.80) | 1.10(0.90-1.20) | 1.56(1.30-1.70) | 2.26(1.80-2.50) | 4.89(2.50-5.06) |
| Week 4 | 9 | 0.32 (0.30-0.80) | 0.38 (0.30-0.80) | 0.70(0.30-0.90) | 0.80(0.40-1.00) | 0.90(0.80-3.30) | 1.46(0.84-3.30) | 2.84(0.88-3.30) |

Abbreviation: TSH, thyroid-stimulating hormone.

**Supplementary** **Table 8 Percentiles of TSH values (μIU/mL) by the time of specimen collection for low birth weight (1500~<2500 g) infants**

| Age, week/day | n | TSH value, μIU/mL (95% CI) | | | | | | |
| --- | --- | --- | --- | --- | --- | --- | --- | --- |
|  |  | 2.5th | 10th | 25th | 50th (Median) | 75th | 90th | 97.5th |
| Week 1 | 6613 | 0.20(0.10-0.20) | 0.40(0.40-0.40) | 0.70(0.70-0.80) | 1.40(1.30-1.40) | 2.30(2.20-2.30) | 3.60(3.50-3.70) | 5.68 (5.50-5.87) |
| 1 | 11 | 0.15(0.00-0.90) | 0.60(0.00-0.90) | 0.85(0.30-1.10) | 1.10(0.80-3.00) | 2.15(1.00-3.65) | 3.60(1.10-3.70) | 3.68(1.30-3.70) |
| 2 | 38 | 0.10(0.10-0.65) | 0.57(0.10-0.97) | 1.03(0.70-1.60) | 2.05(1.30-2.55) | 2.88 (2.30-4.30) | 4.46 (2.99-5.30) | 5.44(4.37-7.10) |
| 3 | 1839 | 0.20(0.20-0.20) | 0.40(0.40-0.50) | 0.80(0.78-0.80) | 1.40(1.40-1.50) | 2.30(2.20-2.40) | 3.56(3.30-3.80) | 5.60(5.10-5.93) |
| 4 | 3603 | 0.12(0.10-0.20) | 0.40(0.40-0.40) | 0.70(0.70-0.80) | 1.40(1.30-1.40) | 2.30(2.20-2.40) | 3.70(3.50-3.90) | 5.90 (5.60-6.20) |
| 5 | 713 | 0.10(0.10-0.20) | 0.30(0.30-0.40) | 0.70(0.60-0.80) | 1.21(1.10-1.40) | 2.20(2.00-2.32) | 3.40(3.10-3.80) | 5.10 (4.72-5.36) |
| 6 | 287 | 0.10(0.10-0.20) | 0.30(0.20-0.40) | 0.70(0.60-0.70) | 1.20(1.00-1.30) | 2.00(1.70-2.20) | 3.10(2.46-3.60) | 4.59(4.07-6.05) |
| 7 | 122 | 0.20(0.20-0.40) | 0.41(0.40-0.60) | 0.80(0.60-1.00) | 1.40(1.10-1.70) | 2.10(1.90-2.30) | 3.06 (2.30-3.33) | 5.29(3.30-6.18) |
| Week 2 | 364 | 0.10(0.02-0.20) | 0.40(0.35-0.50) | 0.60(0.60-0.70) | 1.10(1.00-1.20) | 1.82(1.70-2.10) | 3.10(2.70-3.87) | 7.57(4.90-10.44) |
| Week 3 | 124 | 0.21(0.13-0.40) | 0.43(0.40-0.53) | 0.70(0.60-0.79) | 1.00(0.90-1.20) | 1.53(1.40-1.82) | 3.02(1.90-4.80) | 7.96(4.64-69.10) |
| Week 4 | 23 | 0.15(0.08-0.35) | 0.22(0.10-0.52) | 0.45(0.25-0.65) | 0.70 (0.60-1.20) | 1.25(0.70-2.40) | 2.66(1.18-5.44) | 4.46(1.64-6.00) |

Abbreviation: TSH, thyroid-stimulating hormone.

**Supplementary** **Table 9 Percentiles of TSH values (μIU/mL) by the time of specimen collection for very low birth weight (1000~<1500 g) infants**

| Age, week/day | n | TSH value, μIU/mL (95% CI) | | | | | | |
| --- | --- | --- | --- | --- | --- | --- | --- | --- |
|  |  | 2.5th | 10th | 25th | 50th (Median) | 75th | 90th | 97.5th |
| Week 1 | 1004 | 0.10(0.10-0.10) | 0.30(0.29-0.40) | 0.70(0.60-0.72) | 1.40(1.20-1.50) | 2.40 (2.30-2.60) | 3.72 (3.50-4.10) | 6.09(5.59-6.70) |
| 1 | 3 | 1.64(1.60-2.60) | 1.74(1.60-2.60) | 1.95(1.60-2.60) | 2.30(1.60-2.60) | 2.45(1.60-2.60) | 2.54(1.60-2.60) | 2.59(1.60-2.60) |
| 2 | 10 | 0.37(0.30-1.20) | 0.57(0.30-1.68) | 0.75(0.38-1.90) | 1.85(0.60-2.60) | 2.13(1.65-5.92) | 3.65(1.90-6.80) | 6.01(1.90-6.80) |
| 3 | 208 | 0.10(0.01-0.20) | 0.40(0.20-0.40) | 0.70(0.60-0.80) | 1.27 (1.09-1.50) | 2.40(2.12-2.90) | 3.60(3.20-4.20) | 5.31(4.40-6.40) |
| 4 | 544 | 0.10(0.10-0.14) | 0.30(0.30-0.40) | 0.70(0.66-0.80) | 1.50(1.30-1.60) | 2.53(2.30-2.73) | 4.00(3.60-4.39) | 6.39(5.50-6.93) |
| 5 | 150 | 0.10(0.10-0.17) | 0.20(0.19-0.36) | 0.60(0.40-0.70) | 1.20(1.00-1.46) | 2.30(1.80-2.75) | 3.51(3.10-4.64) | 6.51(4.71-7.20) |
| 6 | 71 | 0.10(0.10-0.20) | 0.30(0.20-0.40) | 0.50(0.30-0.80) | 1.10(0.80-1.40) | 2.05(1.40-2.60) | 2.90(2.24-3.80) | 4.73(3.30-6.40) |
| 7 | 18 | 0.19(0.10-1.04) | 0.65(0.10-1.35) | 1.13(0.42-1.82) | 1.95(1.20-2.50) | 2.50(2.08-3.88) | 3.83(2.50-3.90) | 3.90(3.13-3.90) |
| Week 2 | 123 | 0.30(0.10-0.31) | 0.52(0.30-0.70) | 0.90(0.70-1.20) | 1.60(1.30-1.90) | 2.75(2.30-3.20) | 3.90(3.30-5.00) | 6.79(4.89-7.90) |
| Week 3 | 51 | 0.20(0.10-0.42) | 0.40(0.20-0.60) | 0.80(0.55-1.30) | 1.50(1.20-2.10) | 2.50(2.00-4.70) | 5.40(3.90-7.30) | 8.58(5.40-11.80) |
| Week 4 | 30 | 0.10(0.10-0.32) | 0.29(0.10-0.50) | 0.53(0.30-0.98) | 1.20(0.60-1.50) | 1.60(1.40-2.85) | 3.12(1.61-4.46) | 4.92(2.37-6.80) |

Abbreviation: TSH, thyroid-stimulating hormone.

**Supplementary** **Table 10 Percentiles of TSH values (μIU/mL) by the time of specimen collection for extremely low birth weight (<1000 g) infants**

| Age, week/day | n | TSH value, μIU/mL (95% CI) | | | | | | |
| --- | --- | --- | --- | --- | --- | --- | --- | --- |
|  |  | 2.5th | 10th | 25th | 50th (Median) | 75th | 90th | 97.5th |
| Week 1 | 149 | 0.08(0.00-0.10) | 0.20(0.10-0.30) | 0.50(0.40-0.60) | 1.10(0.80-1.30) | 2.00(1.70-2.40) | 3.60(2.60-4.55) | 5.91(4.50-7.20) |
| 3 | 30 | 0.17(0.10-0.30) | 0.29(0.19-0.40) | 0.43(0.30-0.70) | 0.85(0.55-1.71) | 2.05(1.05-2.65) | 2.72(2.10-3.33) | 3.38(2.55-3.61) |
| 4 | 65 | 0.00(0.10-0.30) | 0.30(0.19-0.40) | 0.50(0.40-0.70) | 0.90(0.70-1.50) | 2.00(1.60-3.70) | 4.34(2.75-5.83) | 6.12(4.78-7.40) |
| 5 | 37 | 0.09(0.03-0.10) | 0.10(0.10-0.30) | 0.30(0.10-0.60) | 1.00(0.40-1.40) | 1.70(1.30-2.40) | 2.54(1.82-4.76) | 4.79(2.50-5.00) |
| 6 | 14 | 0.73(0.70-1.00) | 0.83(0.70-1.23) | 0.98(0.80-1.60) | 1.50(1.05-2.20) | 2.20(1.40-3.60) | 3.51(2.02-7.20) | 6.03(2.20-7.20) |
| 7 | 3 | 0.64(0.60-2.40) | 0.76(0.60-2.40) | 1.00(0.60-2.40) | 1.40(0.60-2.40) | 1.90(0.60-2.40) | 2.20(0.60-2.40) | 2.35(0.60-2.40) |
| Week 2 | 54 | 0.13(0.10-0.53) | 0.53 (0.20-0.90) | 1.10 (0.60-1.63) | 1.85(1.45-2.40) | 3.26(2.38-3.95) | 4.70 (3.51-8.00) | 8.47(4.70-26.00) |
| Week 3 | 31 | 0.10(0.10-0.70) | 0.30(0.10-1.10) | 1.10(0.35-1.65) | 2.00(1.30-3.10) | 3.85(2.60-5.75) | 6.30(4.00-7.40) | 7.63(5.48-8.30) |
| Week 4 | 22 | 0.21(0.10-0.75) | 0.43(0.12-1.00) | 1.00(0.40-1.30) | 1.35(1.00-2.00) | 2.18(1.40-5.42) | 5.77 (2.08-11.17) | 8.92(2.96-11.70) |

Abbreviation: TSH, thyroid-stimulating hormone.


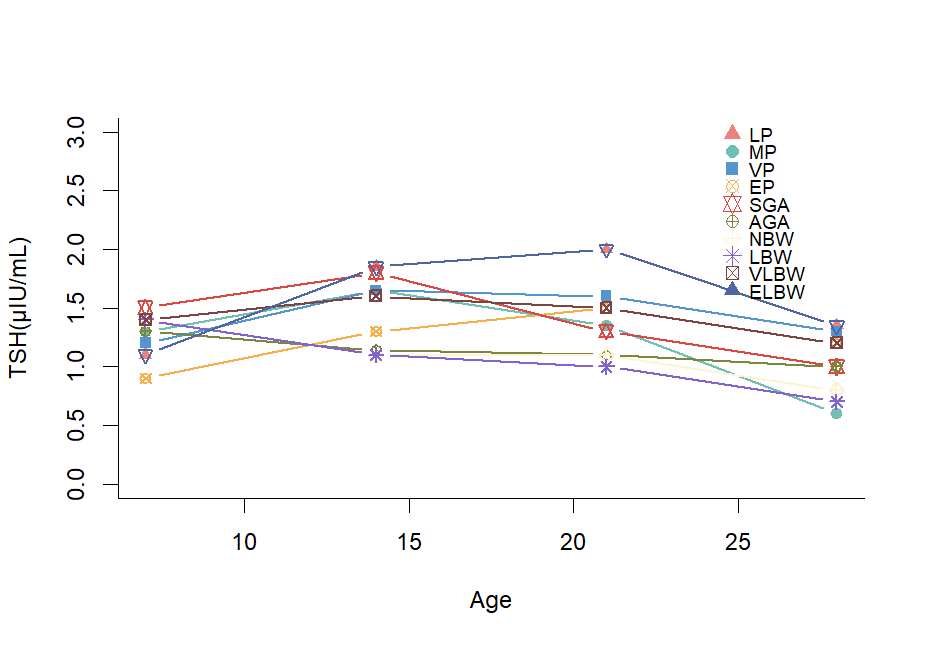


Abbreviation: TSH, thyroid-stimulating hormone.LP, Late preterm.MP, Moderate preterm.VP, Very preterm.EP, Extremely Preterm.NBW, Normal birth weight.LBW, Low birth weight.VLBW, Very low birth weight.ELBW, Extremely low birth weight.SGA, Small for Gestational Age.AGA, Appropriate for Gestational age.

Fig. S1 50th percentile TSH values for preterm infants of different gestational ages, birth weight groups, and SGA and AGA in the first to fourth week of life
